# Acoustic preadaptation to transmit vocal individuality of savanna nightjars in noisy urban environments

**DOI:** 10.1038/s41598-020-75371-4

**Published:** 2020-10-23

**Authors:** Shih-Hsiung Liang, Bruno Andreas Walther, Chia-Hung Jen, Chao-Chieh Chen, Yi-Chih Chen, Bao-Sen Shieh

**Affiliations:** 1 Department of Biotechnology, National Kaohsiung Normal University, Kaohsiung, 824 Taiwan; 2 Department of Biological Sciences, National Sun Yat-Sen University, Kaohsiung, 804 Taiwan; 3 Department of Geography, CDTL, National Kaohsiung Normal University, Kaohsiung, 802 Taiwan; 4 Department of Biomedical Science and Environmental Biology, Kaohsiung Medical University, 100 Shihchuan 1st Road, Kaohsiung, 807 Taiwan; 5 Department of Medical Research, Kaohsiung Medical University Hospital, Kaohsiung, 807 Taiwan

**Keywords:** Ecology, Evolution, Ecology, Environmental sciences

## Abstract

As urbanization has expanded dramatically, the impacts of urban noise on wildlife have drawn increasing attention. However, previous studies have focused primarily on diurnal songbirds and much less on nocturnal nonpasserines such as nightjars. The savanna nightjar has recently successfully colonized urban areas in Taiwan. Using 1925 calls recorded from 67 individuals, we first investigated the individual differences of the acoustic structures; and, for those acoustic variables with significant individual differences, we examined the correlation between the acoustic structures and the ambient noise levels. We then compared the transmission efficacy of vocal individuality among three sets of acoustic variables: all acoustic variables, noise-related variables, and noise-unrelated variables. Using seven artificial frequency-shifted calls to represent seven different individuals in playback-recording experiments, we also investigated the transmission efficacy of vocal individuality and variable accuracy in three different urban noise levels (high, medium, low). We found that all 30 acoustic variables derived from the acoustic structures demonstrated significant individual differences, and 14 frequency-based variables were negatively correlated with ambient noise levels. Although transmission efficacy was significantly affected by urban noise, individuality information was still transmitted with high accuracy. Furthermore, the noise-unrelated structures (which included the maximum frequency, the maximum amplitude frequency, and the mean frequency of the call) had a significantly higher transmission efficacy of vocal individuality than the noise-related variables (which included the minimum frequency, the frequency at the start and the end of the call) in both field observation and playback-recording experiments. We conclude that these noise-unrelated acoustic features may be one of the key preadaptations for this nocturnal nonpasserine to thrive so successfully in its newly adopted urban environment.

## Introduction

The increasing trend of urbanization is a local as well as a global phenomenon^1^. Therefore, urbanization may be one of the most important factors affecting global biodiversity^2^. Consequently, the adaptation of animals to urban environments has become one of the main emerging issues in ecology^3,4^. There are several features of urban areas which are important for birds. In this study, we focus on urban noise. Since acoustic communication is important for their survival and reproduction, including mate attraction, territorial defense, and alarm calls^5^, urban noise is likely to be an important factor shaping the acoustic communication of urban birds^6^.

The majority of urban noise is traffic noise, which is primarily composed of low frequencies in the 0–3 kHz range^7^. Therefore, modification of acoustic signals toward higher frequencies was first documented in a small passerine^8^ and since then in many other bird species^9–11^. However, other authors have questioned the idea that increasing song pitch is an adaptation to noise^12,13^, and some recent studies have argued that a rise in the sound frequency is not adaptive in some species and/or in some contexts^14^. First, a signaling bird might want to transfer information about its aggressive levels and fighting abilities. If this information is more important than just signal detection, a sound shift to a higher frequency in response to urban noise might increase the signal detection at the cost of changing the signal information; that is, higher pitched sounds might indicate less aggressive levels and lower fighting abilities, making them less effective in repelling competitors^15,16^ which, in turn, would be disadvantageous in terms of communication efficacy^17^. Furthermore, information about vocal performance might be associated with song pitch, and if the song pitch increases with the noise levels, the vocal performance may decrease which may result in disadvantages when it comes to mate choice and competition^18,19^.

Second, species-specific factors, including the sound frequency range, body size, and learning ability, have been shown to affect the evolution of the frequency shift adaptation to urban noise. Slabbekoorn and Peet^8^ suggested that species with a sound frequency which is above 2 kHz have no need to increase their sound frequency to avoid urban noise. Hu and Cardoso^20^ further found that species which emit sound frequencies around 1.0–1.5 kHz increased the frequency more than species which emitted a higher or a lower average sound frequency. Parris and McCarthy^21^ investigated the reduction of the active space of bird sounds when moving from quiet to noisy urban areas. They found that the reduction is highest for bird species with a sound frequency around 1.5 kHz and decreases for bird species with a sound frequency higher than 1.5 kHz until it levels off at around 3 kHz. They concluded that bird species with a sound frequency around 1.5 kHz should benefit the most by shifting their sound frequency upward, whereas bird species with a sound frequency higher than 3 kHz should gain little by shifting their sound frequency upward. Therefore, high-frequency species should not need to significantly adjust their sound frequency in response to urban noise, and urban noise might act as an important selective force in filtering those high-frequency species to remain in urban communities^22^. In terms of body size, Roca et al.^14^ demonstrated that smaller-sized species with a sound frequency which overlaps with the prevalent urban noise frequency are more likely to increase their average frequency than larger-sized species which show no evidence of such a shift and may thus depend on other adaptation strategies to cope with the noise. By investigating three passerine families, Billings^23^ found that larger-sized urban species had lower frequency mobbing calls than smaller-sized species in other habitats. As to learning ability, oscines, which learn their songs, showed a stronger positive association between urban noise and song pitch than suboscines, which do not learn their songs^24^; similarly, see Ríos-Chelén et al.^25^ for a lack of evidence of noise-dependent song pitch flexibility in a suboscine. Moseley et al.^26^ suggested that the stronger positive association between urban noise and song pitch in oscines may have resulted from song development in nestlings which tend to learn less masked (higher frequency) songs over masked (lower frequency) songs in noisy situations.

In this study, we wanted to investigate an urban nocturnal nonpasserine which has very different species-specific and context-specific characteristics than most of the bird species which have been investigated so far. We focused on an endemic subspecies of the savanna nightjar (*Caprimulgus affinis stictomus*) which is a nocturnal nonpasserine breeding in Taiwan^27^. The savanna nightjar was recorded as an urban species in Indonesia in the last century^28^ but not in Taiwan until 2000. According to Wang and Lin^29^, the savanna nightjar was rare before 2000 and only occurred in a few administrative areas (three records in 1998). Around 2006, it became abundant in city areas of Taiwan having discovered rooftops as safe breeding sites. After 2010, it had become widespread in lowland urban areas of Taiwan (e.g., distribution map in Wu et al.^30^). The savanna nightjar has not only become a successful urban colonizer but is also an annoying species because the male’s loud territorial calls disturb people during the night^31^. A savanna nightjar produces a territorial call with a sound frequency which is above 2 kHz, produces up to 13,550 calls in one night (our observation), and these loud calls can reach up to an average of 97.7 dB^31^ (measured at the perched spot).

Hence we hypothesize that the calls of male savanna nightjars should show no significant upward frequency shift in response to urban noise because its sound frequency is above 2 kHz. Furthermore, acoustic communication is clearly very important for nocturnal birds^32^. For them to successfully communicate in an urban area, acoustic adaptation to urban noise should thus be critical. Information about the signaler’s identity (its vocal individuality) is transmitted in many nocturnal nonpasserines, such as the scops owl (*Otus scops*)^33^ and the European nightjar (*Caprimulgus europaeus*)^34^. Since identifying neighboring individuals is an adaptation to increase the efficiency of territorial defense, vocal individuality serves an important function in the territorial defense of nocturnal nonpasserines^35^. Given the loudness and sound frequency of its territorial calls, signal detection should not be a problem for this species; rather, transmission efficacy of vocal individuality for territorial defense should be of higher importance. Thus, we also hypothesize that not just signal detection but transmission efficacy of vocal individuality in urban noise should be an important factor for this nocturnal nonpasserine to thrive successfully in its urban environment.

Our objectives were to investigate (1) which acoustic structures of the territorial calls were correlated with urban noise levels, (2) whether the transmission efficacy of vocal individuality was affected by varying urban noise levels, and (3) how transmission efficacy of acoustic structures and individuality, as represented by artificial frequency-shifted calls, was changed among three urban noise levels (high, medium and low) in playback-recording experiments. For this successful urban colonizer, the savanna nightjar, we expect that (1) acoustic structures of their calls are less affected by urban noise, that is, most acoustic structures are not correlated with urban noise levels; and (2) the transmission efficacy of vocal individuality based on the noise-unrelated structures can remain high in all urban noise levels.

Using individuals as sampling units, we recorded territorial calls as well as the ambient noise levels within each individual’s territory. To sample as many different individuals as possible and to avoid repeated sampling, we recorded the territorial calls of the savanna nightjar in eight areas of Taiwan spread from north to south. Because any differences in acoustic structures could be related to urban noise levels but could also be due to geographic variation, we first performed a principal component analysis (PCA) to examine any possible geographic patterns of the calls. If there is no distinct boundary of calls of individuals from the eight different geographic areas, we could then treat all the sampled individuals as one population and pool them for further analysis.

The transmission efficacy of vocal individuality is defined for the purposes of our study as ‘the accuracy of acoustic structures to transmit individual identity information through the environment’. Acoustic structures with high transmission efficacy of vocal individuality must not only encode individual information but this information must also be transmitted through the ambient environments to the receiver with a high accuracy. Acoustic structures with significant individual differences are more likely to encode individual information than those without significant individual differences. Therefore, we used calls as sample units and individuals as groups. We first identified those acoustic structures which can be used by nightjars to identify individuals. We surmised that those structures with significant individual differences have a high potential for encoding individual information, and are therefore used for individual identity. Correlating the acoustic structures with the ambient noise levels allowed us to classify acoustic variables into either noise-related variables (with significant correlations) or noise-unrelated variables (with non-significant correlations). In order to demonstrate how urban noise affected the transmission efficacy of vocal individuality, we compared three sets of acoustic variables: all acoustic variables, noise-related variables, and noise-unrelated variables. We used the accuracy value (1—misclassification rate) calculated from a discriminant function analysis (DFA) to assess the transmission efficacy of vocal individuality. The purpose of the DFA is to discriminate all sampled individuals based on the different sets of acoustic variables. Higher accuracy values are assumed to indicate higher transmission efficacy of individual information through those acoustic structures (see “Statistical analyses” in the “Methods” for details). To verify the comparative results of our field observations, we also generated seven artificial calls with different frequency shifts (− 300 Hz, − 200 Hz, − 100 Hz, 0, + 100 Hz, + 200 Hz, + 300 Hz) using a good quality call to represent seven different artificial individuals and conducted playback-recording experiments to compare the transmission efficacy of individuality for noise-related variables and noise-unrelated variables in three urban noise levels (high, medium, and low).

## Results

### Geographic variation of the savanna nightjar calls

**Figure 1:**
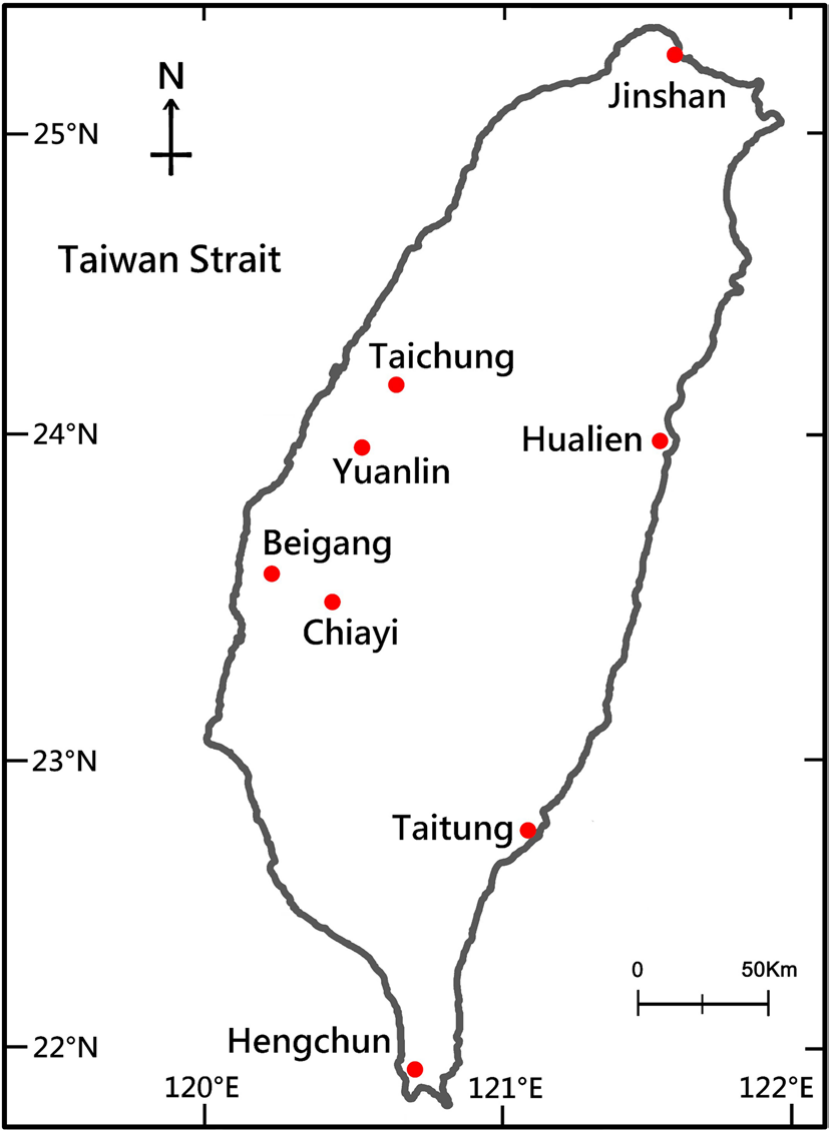
A map of the eight sampling areas in Taiwan. Sampling areas are marked with red dots.

**Figure 2:**
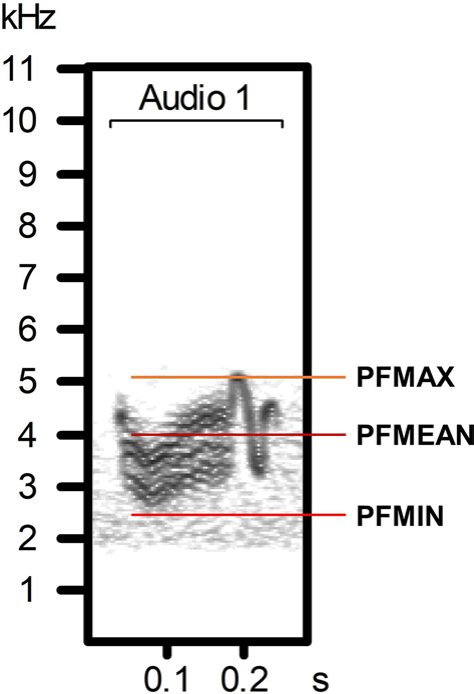
The spectrogram for one representative call of a savanna nightjar. The spectrogram was produced using the Avisoft-SASLab Pro software with the following spectrogram parameters: sampling frequency = 22.05 kHz, FFT = 512, and hamming window. The representative call can be heard in Supplementary Audio S1. *PFMAX* the maximum frequency of the call, *PFMEAN* the mean frequency of the call, *PFMIN* the minimum frequency of the call.

We recorded a total of 1925 calls from 67 individuals in eight areas of Taiwan (Fig. 1). The spectrogram for one representative call can be seen in Fig. 2 and be heard in Supplementary Audio S1. The recorded 67 individuals had calls with a minimum frequency of 2275.05 ± 28.14 Hz (mean ± SE), a mean frequency of 3788.5 ± 23.59 Hz, and a maximum frequency of 5348.34 ± 53.27 Hz (see Table 1 for variable description and see Supplementary Table S1 for descriptive statistics of all 30 acoustic variables).

**Table 1:** Descriptions of 30 acoustic variables. Noise-related variables (P < 0.05) are shown into bold italics.

Variable	Description (unit)
**Time-based variables**	
DUR	Duration of the call (s)
DISTTOMAX	Temporal distance from start to the location of the maximum amplitude (s)
**Frequency-based variables**	
*PFSTART*	At start of the call, peak frequency (the frequency of the maximum amplitude) is measured (Hz)
*Q1START*	At start of the call, the quartile 25% frequency (below this frequency is 25% of the total energy) is measured (Hz)
*Q2START*	At start of the call, the quartile 50% frequency (below this frequency is 50% of the total energy) is measured (Hz)
*Q3START*	At start of the call, the quartile 75% frequency (below this frequency is 75% of the total energy) is measured (Hz)
*PFEND*	At end of the call, peak frequency is measured (Hz)
*Q1END*	At end of the call, the quartile 25% frequency is measured (Hz)
*Q2END*	At end of the call, the quartile 50% frequency is measured (Hz)
*Q3END*	At end of the call, the quartile 75% frequency is measured (Hz)
PFMAXA	At the location of the maximum amplitude of the call, peak frequency is measured (Hz)
Q1MAXA	At the location of the maximum amplitude of the call, the quartile 25% frequency is measured (Hz)
Q2MAXA	At the location of the maximum amplitude of the call, the quartile 50% frequency is measured (Hz)
Q3MAXA	At the location of the maximum amplitude of the call, the quartile 75% frequency is measured (Hz)
*PFMIN*	The minimum frequency of the call; the lowest peak frequency of the call (Hz)
*Q1MIN*	The minimum quartile 25% frequency of the call (Hz)
*Q2MIN*	The minimum quartile 50% frequency of the call (Hz)
Q3MIN	The minimum quartile 75% frequency of the call (Hz)
PFMAX	The maximum frequency of the call (Hz); the highest peak frequency of the call
*Q1MAX*	The maximum quartile 25% frequency of the call (Hz)
Q2MAX	The maximum quartile 50% frequency of the call (Hz)
Q3MAX	The maximum quartile 75% frequency of the call (Hz)
PFMEAN	The mean frequency of the call (Hz); the mean peak frequency of the call
*Q1MEAN*	The mean quartile 25% frequency of the call (Hz)
*Q2MEAN*	The mean quartile 50% frequency of the call (Hz)
Q3MEAN	The mean quartile 75% frequency of the call (Hz)
**Frequency-modulation-based variables**	
*PFSTDDEV*	The relative standard deviation (= standard deviation/mean value) of the peak frequency within the call; frequency modulation within the call
Q1STDDEV	The relative standard deviation (= standard deviation/mean value) of the quartile 25% frequency within the call
Q2STDDEV	The relative standard deviation (= standard deviation/mean value) of the quartile 50% frequency within the call
Q3STDDEV	The relative standard deviation (= standard deviation/mean value) of the quartile 75% frequency within the call

**Figure 3:**
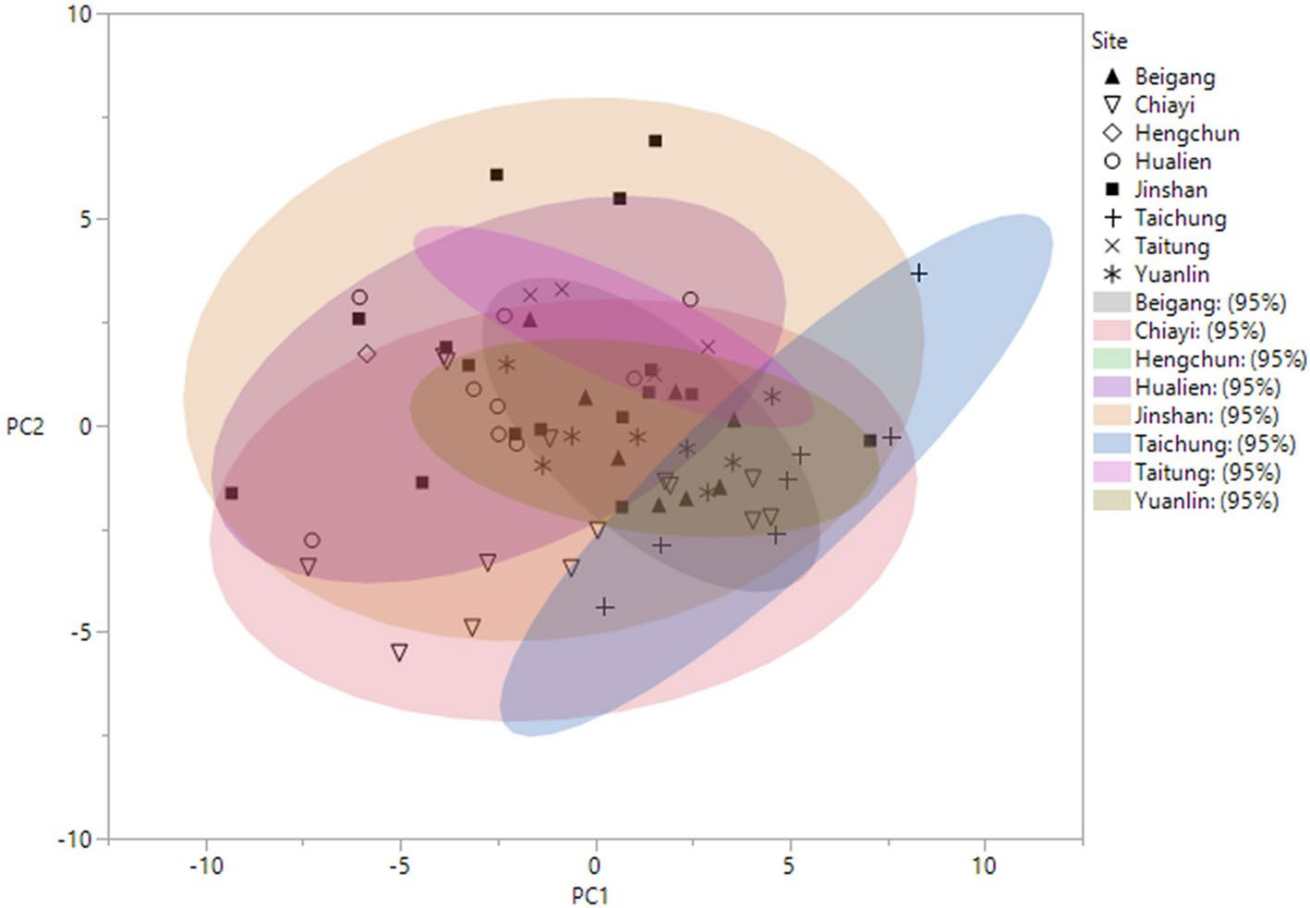
A bi-dimensional plot of the PCA. The PCA was performed on the 30 acoustic variables derived from the calls of 67 individuals from eight areas of Taiwan (see Supplementary Table S2 for information of PC1 and PC2). The 95% confidence ellipses of the groups of individuals from different geographic areas are shown on the plot.

Five principal components were derived from the 30 normalised acoustic variables (Supplementary Table S2). The first two components (PC1 and PC2) accounted for 65.7% of the total variation. PC1 accounted for 45.7% of the total variation, with a higher value of PC1 corresponding to a lower mean frequency distribution (PFMEAN, Q1MEAN, Q2MEAN, and Q3MEAN). PC2 accounted for 20.0% of the total variation, with a higher value of PC2 corresponding to a higher minimum frequency (PFMIN) and a lower frequency modulation within the call (PFSTDDEV). When we produced 95% confidence ellipses of groups of individuals from different areas on the plot of PC1 against PC2, ellipses of seven areas overlapped, and the single individual point recorded from the Hengchun area was located within the ranges of Jinshan, Hualien, and Chiayi (Fig. 3). Since we found no distinct boundaries among the individuals from the eight different areas, we pooled all individuals together for further analyses.

### Acoustic variables correlated with ambient noise level

Among these 30 acoustic variables, 15 variables (denoted noise-related variables) had significant correlations with the ambient noise levels while the other 15 variables (denoted noise-unrelated variables) did not (Spearman rank tests in Supplementary Table S3). Every one of the 15 noise-related variables involved sound frequency, and 14 of these demonstrated a negative correlation with ambient noise levels; only the frequency modulation variable (PFSTDDEV) showed a positive correlation with ambient noise levels (Supplementary Table S3). The noise-unrelated variables included two time-based variables, 10 frequency-based variables, and three frequency modulation-based variables (Supplementary Table S3).

### Ambient noise levels and the transmission efficacy of vocal individuality

All 30 acoustic variables demonstrated significant individual differences (Kruskal–Wallis tests in Supplementary Table S4); therefore, we included all 30 variables into the DFA. The overall accuracy value of the DFA using all 30 variables was 84.6% with a 95% biased-corrected confidence interval (BCI) of 82.3%–85.0% for 20,000 bootstrap replications. The overall accuracy value of the DFA using only the 15 noise-unrelated variables decreased to 75.9% with a 95% BCI of 72.2%–77.3%, whereas the overall accuracy value of the DFA using the 15 noise-related variables dropped even lower to 51.8% with a 95% BCI of 48.0%–51.6%.

**Figure 4:**
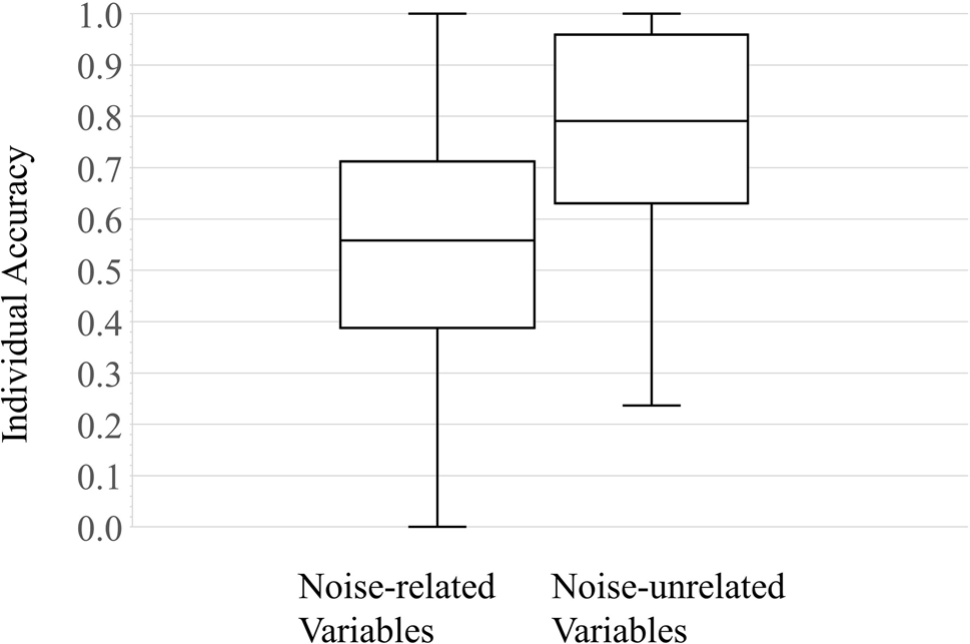
A box plot of comparison of individual accuracy values. Individual accuracy values of the DFA using the 15 noise-unrelated variables were compared with those of the DFA using the 15 noise-related variables (Wilcoxon signed rank test, n = 67, W = 958, P < 0.0001).

**Figure 5:**
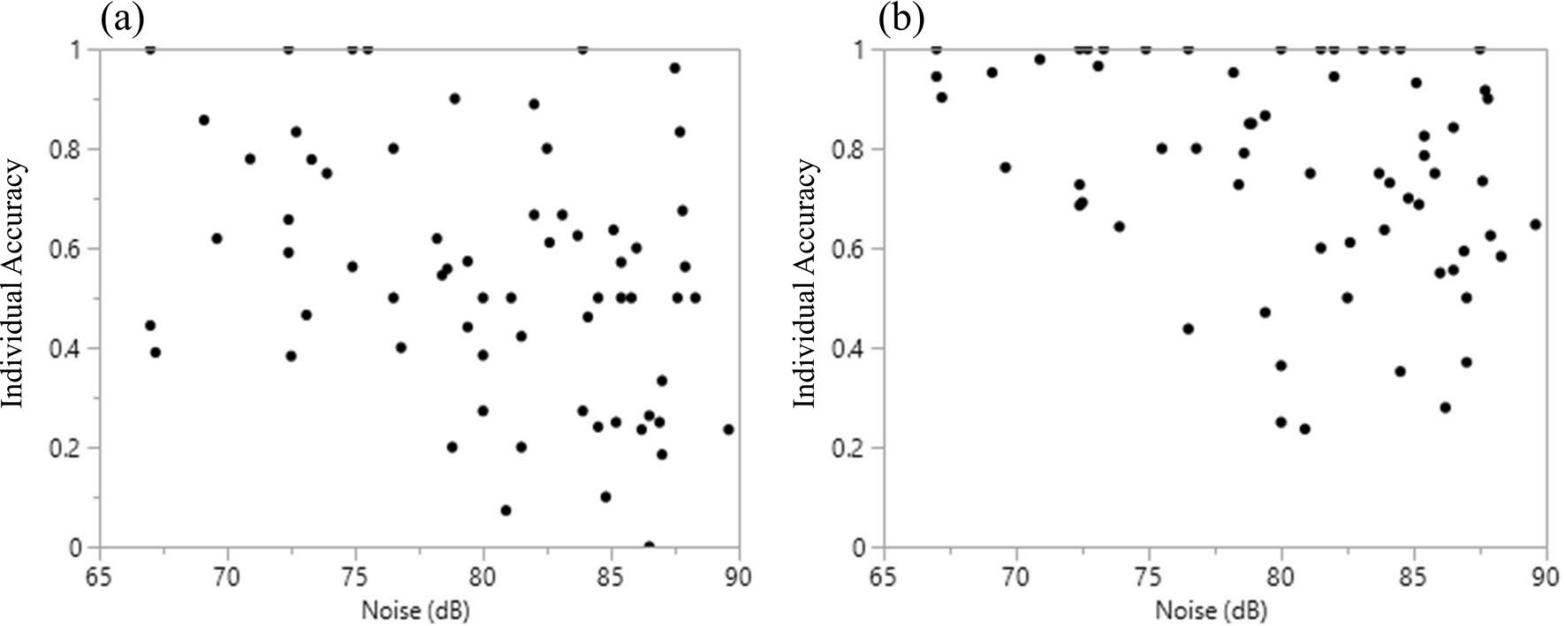
Plots of individual accuracy values against ambient noise levels. The individual accuracy values derived from DFA using (**a**) noise-related variables or (**b**) noise-unrelated variables were significantly decreased as the ambient noise levels increased (noise-related variables: Spearman ρ = − 0.323, P = 0.009; noise-unrelated variables: Spearman ρ = − 0.347, P = 0.005) (n = 65 individuals because noise measurements were not taken for two individuals).

Individual accuracy values of the DFA using the 15 noise-unrelated variables were significantly higher than those of the DFA using the 15 noise-related variables (Wilcoxon signed rank test, n = 67, W = 958, P < 0.0001) (Fig. 4). Furthermore, the individual accuracy values using noise-related variables or noise-unrelated variables significantly decreased as the ambient noise levels increased (noise-related variables: Spearman ρ = − 0.323, P = 0.009; noise-unrelated variables: Spearman ρ = − 0.347, P = 0.005) (Fig. 5). Ambient noise levels varied from 67.0 dB in the ‘quietest’ recording sites to almost 89.6 dB in the ‘loudest’ sites.

**Figure 6:**
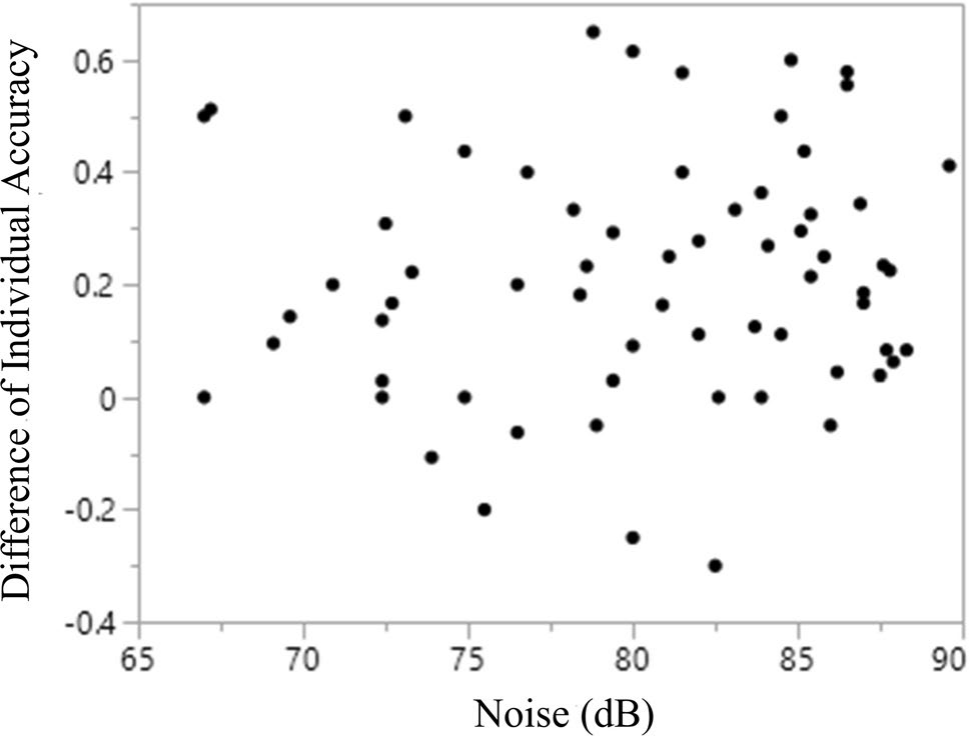
A plot of the differences of individual accuracy values against ambient noise levels. Differences, which was taken as the accuracy value of the DFA using the noise-unrelated variables minus the accuracy value of the DFA using the noise-related variables from the same individual, were not significantly correlated with ambient noise levels (Spearman rank correlation coefficient ρ = 0.097, P = 0.443) (n = 65 individuals because noise measurements were not taken for two individuals).

To test if the trends in Fig. 5 are similar or dissimilar, we took the difference, which was taken as the accuracy value of the DFA using the noise-unrelated variables minus the accuracy value of the DFA using the noise-related variables from the same individual, taken from Figs. 5a and b. We found no significant trend for the differences with ambient noise levels (Spearman ρ = 0.097, P = 0.443) (Fig. 6).

### Transmission efficacy in playback-recording experiments

The overall accuracy value of the DFA using all 29 variables (deleting the fixed variable in this experiment, namely duration) for the seven artificial individuals with different frequency-shifted calls in playback-recording experiments was 100% in all three urban noise levels (ambient noise level, high: mean = 83.7 dB, range = 79.3–89.6 dB; medium: mean = 74.6, range = 73.1–76.3 dB; low: mean = 71.4 dB, range = 69.9–74 dB). The overall accuracy value of the DFA using only the three noise-unrelated variables (PFMAXA, PFMAX, PFMEAN) decreased to 71.4% with a 95% BCI of 51%–81.7% for 20,000 bootstrap replications in high urban noise level, 85.7% with a 95% BCI of 69.4%–87.8% in medium urban noise level, and 81.6% with a 95% BCI of 59.2%–85.7% in low urban noise level, respectively. The overall accuracy value of the DFA using only the three noise-related variables (PFSTART, PFEND, PFMIN) dropped significantly lower (than those of the DFA using the three noise-unrelated variables) to 30.6% with a 95% BCI of 10.2%–36.7% in high urban noise level, 59.2% with a 95% BCI of 34.7%–69.4% in medium urban noise level, and 38.8% with a 95% BCI of 18.4%–42.9% in low urban noise level, respectively.

**Figure 7:**
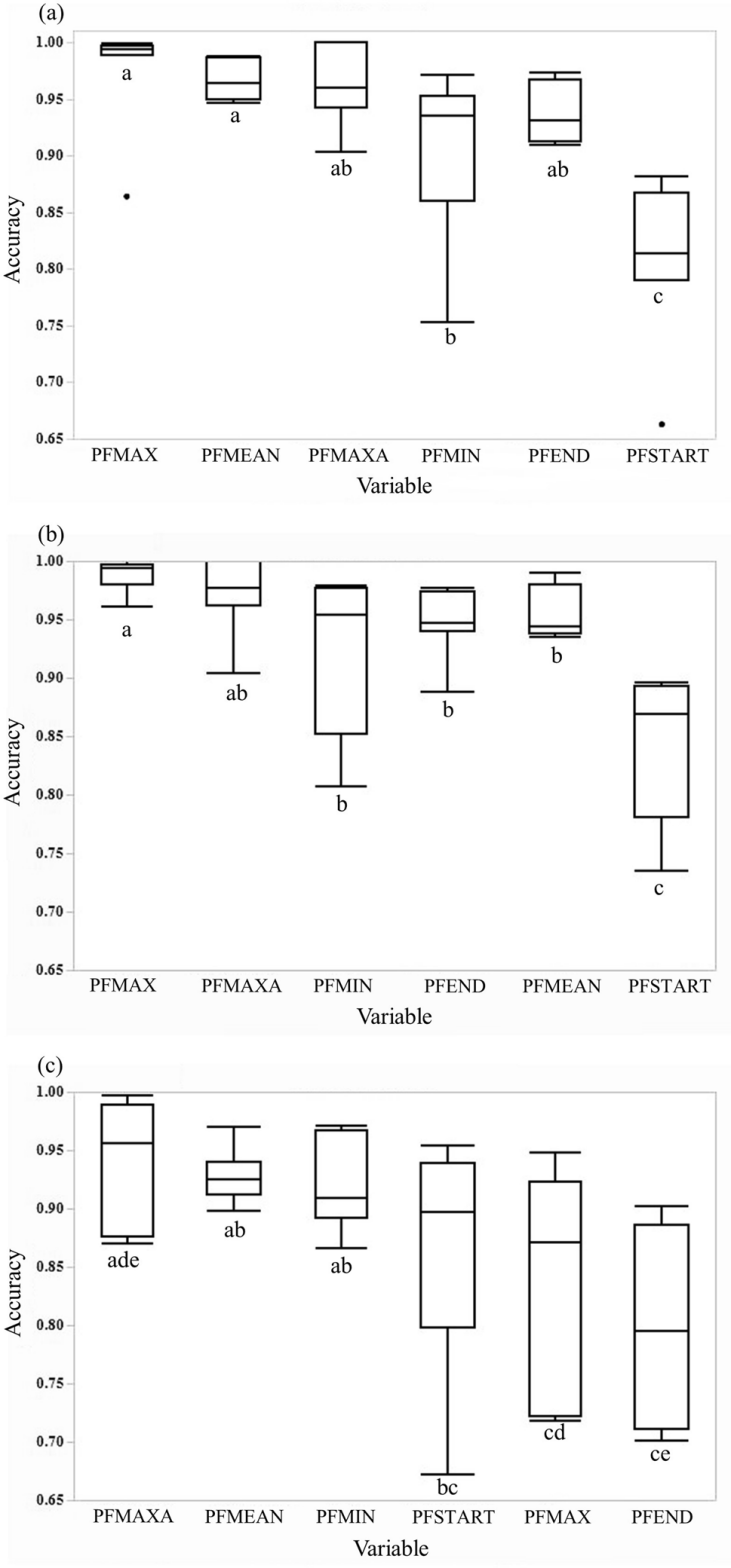
Box plots of comparison of variable accuracy values in different urban noise levels: (**a**) low; (**b**) medium; and (**c**) high. Different letters indicate significant difference for paired comparison (see Supplementary Table S5 for all the multiple comparison results using Wilcoxon signed rank tests).

We compared the measurements on spectrograms of broadcast call recordings (B) and received call recordings (R) to calculate the variable accuracy values, defined as 1 − (|R – B|/B), for the three noise-related variables (PFSTART, PFEND, PFMIN) and the three noise-unrelated variables (PFMAXA, PFMAX, PFMEAN). In all three noise levels (high, medium, and low), the variable accuracy values were significantly different among the six variables (Friedman rank test with frequency-shifted calls as blocks, df = 5, high urban noise level: ChiSquare = 22.8, *P* = 0.0004; medium urban noise level: ChiSquare = 23.2, *P* = 0.0003; low urban noise level: ChiSquare = 13.5, *P* = 0.0189). Furthermore, in low urban noise level, the two noise-unrelated variables, PFMAX and PFMEAN had significantly higher variable accuracy values than those of the two noise-related variables, PFMIN and PFSTART (Wilcoxon signed rank test, *P* < 0.05) (Fig. 7) (see Supplementary Table S5 for all the multiple comparison results). In medium urban noise level, the noise-unrelated variable, PFMAX had significantly higher variable accuracy values than those of the three noise-related variables, PFSTART, PFEND, PFMIN (Wilcoxon signed rank test, *P* < 0.05). In high urban noise level, the noise-unrelated variable, PFMAXA had the highest median accuracy value among variables, and only this noise-unrelated variable had significantly higher accuracy values than that of the noise-related variable, PFSTART; none of the noise-unrelated variables had accuracy values significantly higher than all of the three noise-related variables (Fig. 7).

## Discussion

Increasing numbers of bird species have been observed to survive successfully in urban environments^36,37^. In Taiwan, the savanna nightjar has become one of the most successful urban residents^38^. To better understand their acoustic adaptation to noisy urban environments, we investigated the acoustic structures of individual males’ territorial calls using two proposed hypotheses (see “Introduction” for details): no frequency shifting and transmission efficacy of vocal individuality.

We hypothesized that the savanna nightjar should not demonstrate an upward frequency shift of their calls in response to the urban noise based on the species characteristics (sound frequency above 2 kHz, and nonpasserines do not possess a significant learning ability to acquire their call structures). Consequently, we predicted that the frequency-based variables of the calls should have no significant correlations with ambient noise levels. However, our findings showed two surprising trends. First, those acoustic variables (or structures) of the territorial calls which were significantly correlated with ambient urban noise levels were all related to frequency. Second, and more importantly, the correlations were significantly negative instead of positive or neutral except for the frequency modulation variable (PFSTDDEV). That is, as the ambient noise levels increased, sound frequency features, including the frequency at the start and the end of the call, and the minimum frequency of the call decreased (Supplementary Table S3). These negative correlations are quite the opposite to the results of most, if not all, previous studies of songbirds in which the ambient noise levels and the minimum song frequency were positively correlated^9,39^.

Furthermore, we also hypothesized that not just signal detection but transmission efficacy of vocal individuality in urban noise should be important for our study species. For nocturnal birds, vocal individuality is a common feature^40,41^ and is critical for territorial defense^5^. Acoustic structures with high transmission efficacy of vocal individuality must not only encode individual information but this information must also be transmitted through the ambient environments to the receiver with a high accuracy. We assessed the transmission efficacy of vocal individuality with accuracy values derived from a DFA. If the accuracy values generated by a DFA are low, it could be due either to a noisy environment which erodes the individual information during transmission and/or because the acoustic variables do not encode enough individual information for individual recognition. In our quietest recording sites with noise levels ranging from 67 to 69 dB, the individual accuracy values were > 94% using all acoustic variables. This is similar to the overall accuracy value of 94.5% which Chang et al.^40^ found in their study of the large-tailed nightjar (*Caprimulgus macrurus*) in a very quiet forest and plantation environment. Consequently, we are confident that the 30 variables which we used did encode enough individual information for recognition.

As to the noise erosion, we found that the accuracy values (transmission efficacy) did decrease with the increase of noise levels (Fig. 5). In our field study, our mean noise level was 80.24 dB (Supplementary Table S1) which is a typical ‘loud’ noise level for an urban environment in Taiwan. Nevertheless, the overall accuracy value of DFA using all 30 acoustic variables remained at 84.6%. When we divided these 30 variables into two categories, we found that the nightjar’s calls consisted of two categories of acoustic structures (15 noise-unrelated and 15 noise-related variables). Our overall accuracy values remained high for the noise-unrelated variables (75.9%) despite our recordings being done in a noisy environment, but decreased for the noise-related variables (51.8%). Furthermore, individual accuracy values were significantly higher in DFAs using noise-unrelated variables than using noise-related variables. Therefore, based on the equal number of 15 variables each, we conclude that the noise-unrelated variables in calls of the savanna nightjar had a significantly higher transmission efficacy of vocal individuality despite being received in our noisy urban environment.

Our results of playback-recording experiments also verified that noise-unrelated structures which included (1) the frequency at the location of the maximum amplitude of the call (PFMAXA), (2) the maximum frequency of the call (PFMAX), and (3) the mean frequency of the call (PFMEAN), showed significantly higher transmission efficacy of individuality than noise-related structures which included (1) the frequency at the start of the call (PFSTART), (2) the frequency at the end of the call (PFEND), and (3) the minimum frequency of the call (PFMIN) in all three urban noise levels.

In the present study, noise-related acoustic structures included the frequency at the start and the end of the call, and the minimum frequency of the call. We showed that these noise-related acoustic structures have a high potential of encoding individual information because of the statistically significant differences which we detected between individuals. However, lower frequency calls from the same individual might be emitted to indicate higher levels of aggression^15,42^. In support of this interpretation, Cardoso^43^ showed that nonpasserines use lower-frequency sounds to demonstrate higher aggressive intent and greater motivation to attack an individual. Therefore, the minimum frequency of the nightjar calls might vary with aggressive intent for the same individual and consequently encode less accuracy in identifying individuals. Thus, this flexibility in encoding individual aggressive intent might result in the lower accuracy values of the DFA using the noise-related variables in our “quietest” noisy environments.

As for the eroding problem, we generated seven frequency-shifted calls to represent seven different individuals; the results of our playback-recording experiments on those seven frequency-shifted calls confirmed that the three noise-unrelated structures (PFMAXA, PFMAX, PFMEAN)) have significantly higher transmission efficacy of individuality than the three noise-related structures (PFSTART, PFEND, PFMIN) in all three urban noise levels (83.7 dB, 74.6 dB, and 71.4 dB). The seven frequency-shifted calls were generated by shifting the frequency domain of the same original call to different frequency ranges (− 300 Hz, − 200 Hz, − 100 Hz, 0, + 100 Hz, + 200 Hz, + 300 Hz) without changing the temporal pattern, and the minimum frequency of all the generated calls were controlled to be above the 2000 Hz. Because these generated frequency-shifted calls had significantly different frequency-based structures to represent seven different artificial individuals, the lower transmission efficacy of individuality for the three noise-unrelated structures could not have resulted from the encoding problem of those frequency-based structures but must be due to the eroding problem. Thus, we conclude that the noise-unrelated structures had better transmission efficacy than that of noise-related structures because they were less eroded by urban noise.

Furthermore, our results of comparing the transmission accuracy among variables in the playback-recording experiments demonstrated that the noise-unrelated structures, e.g., the maximum frequency of the call (PFMAX), performed significantly better in transmission accuracy than the noise-related structures, e.g., the minimum frequency of the call (PFMIN) and the frequency at the start of the call (PFSTART) in low (71.4 dB) and medium (74.6 dB) urban noise levels. The maximum frequency of the call (PFMAX, 5348.34 ± 53.27 Hz in Supplementary Table S1) in the savanna nightjar was much higher than the usual traffic noise (mean maximum frequency ± SE = 1202.7 ± 117.6 Hz) measured in Taiwan in a previous study^44^. Therefore, once nightjars entered the urban environment, with the main source of noise being the traffic noise, noise-unrelated acoustic structures were less masked by the traffic noise and thus were less eroded by the traffic noise; as a result, they have a higher transmission accuracy than noise-related acoustic structures.

Although noise-unrelated structures demonstrated significantly higher transmission efficacy than noise-related structures, the difference of the transmission efficacy between noise-unrelated structures and noise-related structures were independent of noise levels (Fig. 6). This might be because the analyses of our study were performed using time–frequency based variables, and the main source of noise in our study areas was mainly the traffic noise, which might have similar time–frequency patterns and thus similar effects regardless of the noise levels. Thus, we suggest that the simple fixed calls of the savanna nightjar, with their loudness and their noise-unrelated acoustic structures, particularly much higher maximum frequency than the traffic noise, can provide high transmission efficacy of vocal individuality even in urban noise.

We found a negative correlation between the ambient noise levels and the minimum sound frequency, which is a somewhat unexpected result. We therefore propose a novel hypothesis. Specifically, we suggest that the artificial night lighting present in urban environments may be the other important driving factor which is confounded with the urban noise and which indirectly affects acoustic structures. In our study, we usually recorded the calls of the savanna nightjars at road intersection which have not only high levels of noise but also very bright artificial lights which attract phototactic insects^45^ which consequently become hotspots for a nightjar’s foraging. For nightjars, urban lights at night not only provide benefits because of the dense aggregations of insect prey but also because of the improved visibility to better catch these flying insects^46^. Therefore, territorial defense is likely intense around these hotspots of light and food. The lower frequency calls emitted at noisier sites might indicate the stronger competitive ability/larger body size^47^ or higher levels of aggression^15,42^ of those territorial males which occupy these hotspots. In other words, we hypothesize that those territories with higher urban noise levels and brighter artificial lights were occupied by stronger/larger males or more aggressive males which also emit lower frequency calls.

Our findings showed that the correlations between the 14 sound frequency features of the savanna nightjar calls and the ambient noise levels were significantly negative. Although transmission efficacy was affected by urban noise, individuality information of the call was transmitted with high accuracy because of the noise-unrelated acoustic structures which included the maximum frequency of the call, the maximum amplitude frequency and the mean frequency of the call. We concluded that these noise-unrelated acoustic features of the calls in the savanna nightjar may be one of the key preadaptations for this nocturnal nonpasserine to thrive and expand so successfully in urban environments in such a short period of time (less than one decade).

## Methods

### Study area and field observations

We recorded the territorial calls of male savanna nightjars in eight areas of Taiwan, from north to south: Jinshan District (16 nightjars), Taichung City (7 nightjars), Hualien City (9 nightjars)*,* Yuanlin City (8 nightjars), Beigang Township (8 nightjars), Chiayi City (14 nightjars), Taitung City (4 nightjars), and Hengchun Township (1 nightjar) (Fig. 1) during April-June of 2018*.* Sound recordings were collected in the downtown of each area using a Denon Portable IC Recorder (DN-F20R, sampling rate = 48 kHz, 16 bit, wav format) equipped with a Sennheiser ME67 unidirectional microphone. We made recordings between 19:00 to 24:00 in good weather during 1–2 consecutive nights for each area. If we recorded in the same area for more than one consecutive nights, the recording range of the second night was at least 1000 m away from the previous recording range. The maximum territory size of the savannah nightjar observed by Chan^48^ in urban areas of Taiwan was 83,424 m^2^ with a radius of about 163 m. Therefore, we are confident that we avoided recording the same territorial male twice. Territorial calls from an individual were recorded until the individual stopped calling or flew out of our recording range. When emitting territorial calls, nightjars either perched on some artificial structure, such as antennas or fences on roof tops, or they flew around the tops of buildings. Because the loud territorial calls recorded in these two situations demonstrate the same time–frequency patterns on spectrograms and sound the same when listened to, we did not differentiate between them while recording. We always attempted to record at the closest possible distance to the calling individual by moving closer to the individual at the street level.

After measuring the individual’s calls, we immediately took three samples of the maximum noise levels (maximum hold function, C-weighting function on Sound Level Meter TES-1350, TES, Taiwan) near the calling location and close to a road intersection within the calling range following the procedures detailed in Shieh et al.^49^. During each noise sampling, the sound level meter was held horizontally at a height of about 1.5 m and turned 360° clockwise to measure the maximum noise level from all directions within a time period of about 30 s. We then averaged the three samples of maximum noise measurements to arrive at our value of ‘ambient noise levels’ for each individual. Therefore, ambient noise levels were measured at a height of 1.5 m, which was not only the height at which anthropogenic sources such as traffic and human activity are the main sources of noise but also the height at which we held the microphone to record the nightjar calls emitted at a height of usually more than 10 m.

### Playback-recording experiments

Seven artificial calls were generated using the frequency shift function (+ 300 Hz, + 200 Hz, + 100 Hz, 0 Hz, − 100 Hz, − 200 Hz, − 300 Hz) under the Frequency Domain Transformations tool in Avisoft-SASLab Pro software v5.2.12 from a source call with good quality (see Supplementary Table S6 for descriptions of acoustic measurements) and after band-pass filters (2–7 kHz) for noise removal. These seven artificial frequency-shifted calls represented seven different artificially created individuals and were identified by their frequency shift values (+ 300 Hz, + 200 Hz, + 100 Hz, 0 Hz, − 100 Hz, − 200 Hz, − 300 Hz). We then copied each frequency-shifted call 10 times with equal silent intervals and same amplitude as a group, and then we merged the seven groups of frequency-shifted calls into one sound file with alternated orders following a Latin square design. A total of seven merged sound files were obtained, and each was broadcast once in three sites of different urban noise levels: high, medium and low. The playback-recording experiments were conducted at three sites near or on the campus of Kaohsiung Medical University (22.648 N, 120.310 E). We also took 10 samples of the maximum noise level (maximum hold function, C-weighting function on Sound Level Meter TES-1350, TES, Taiwan) at the recording sites during the playback periods to obtain the ambient noise levels for each site. The first site was on the traffic roadside near a road intersection and had the highest ambient noise levels with a mean of 83.7 dB (n = 10) and a range of 79.3–89.6 dB. The second site was on the sport field of the campus about 30–50 m away from the traffic road and had the medium ambient noise levels with a mean of 74.6 (n = 10) and a range of 73.1–76.3 dB. The third site was on a 4^th^ floor roof garden of the campus and had the lowest ambient noise levels with a mean of 71.4 dB (n = 10) and a range of 69.9–74 dB.

We used a Denon Portable IC Recorder (DN-F20R) connected to a speaker (Sony SRS-X11) for the playback experiments. The speaker was placed 1.5 m above the ground on a tripod. Playback-recording experiments were conducted between 17:00 and 18:30 h, a period with high traffic levels. The seven merged sound files were played back at a standardised volume with a sound-pressure level of 82.5 dB at 1 m from the speaker, and about 62.5 dB at 10 m from the speaker. The sound-pressure level of the broadcasting sound which we received at 10 m is about the same amplitude that we recorded a nightjar call at a distance of 28.8 m away from its calling spot with an amplitude of 97.7 dB. We recorded with a Denon Portable IC Recorder (DN-F20R) connected to a shotgun microphone (Sennheiser ME67) that was placed on a stand 1.5 m above the ground and oriented toward the broadcasting speaker at a distance of 10 m. All the recordings were set at the same recording levels and same settings (sampling rate = 48 kHz, 16 bit).

### Sound analyses

We selected high-quality calls with clear acoustic structures on spectrograms and thus excluded any recordings with low-quality calls, which were, for example, emitted when the calling nightjar was too far away, or its calls were overlapped with calls from neighboring nightjars.

We also excluded any recordings where the individual only uttered one call (two calls being the minimum needed for inclusion). This left us with a total of 1925 calls from 67 individuals for our analyses, with a mean of 28.7 ± 3.1 calls/individual and a range of 2–97 calls/individual. The band-pass filters were set from 2.0–6.8 kHz and adjusted for each individual to reduce noise components. Using the recordings, we produced spectrograms with the following spectrogram parameters in the software: sampling frequency = 22.05 kHz, FFT = 512, hamming window, frequency resolution = 43 Hz, and time resolution = 2.9 ms. We quantified 30 acoustic variables (Table 1) from the spectrograms using the Automatic Parameter Measurements setup in the Avisoft-SASLab Pro software v5.2. We marked a call with the section label manually on the spectrogram by eye, and then two time-based parameters were measured (duration of the call and the temporal distance from the start to the location of the maximum amplitude) (see the manual^50^ of the Avisoft-SASLab Pro software for details). To automatically measure parameters other than the time-based on the labelled section, we specified four spectrum-based parameters (peak frequency, quartile 25%, quartile 50% and quartile 75%) to be measured at seven locations of the labelled section (start, end, maximum amplitude of the call, minimum parameter of entire call, maximum parameter of entire call, mean parameter of entire call, relative standard deviation of entire call); thus, 24 frequency-based parameters and four frequency-modulation-based parameters were automatically measured based on the labelled sections on the spectrograms.

For the playback-recording experiments, the recordings were first analyzed using the same settings as the above except for two differences. We adjusted the band-pass filters to 2.0–7.3 kHz because of the high frequency shift value (up to 300 Hz). Furthermore, we used the duration of the source call as the duration of all received calls; that is, we fixed the duration of the call section while marking, and the other 29 variables were automatically measured on the marked section by the software to reduce any possible human measurement errors^51^.

### Statistical analyses

To examine possible geographic variation of the calls, we used individuals as our sample units (n = 67), and the averaged measurements from the calls of each individual were analyzed using a PCA. The PCA was performed on the 30 acoustic variables after a normalised transformation of each variable to a mean of zero and unit standard deviation (software PRIMER 6, version 6.1.5). We retained the five components with eigenvalues greater than one and interpreted each component based on its correlations with the original variables. However, because there is a sharp decrease of the eigenvalue and of the explained variance to smaller values from PC2 to PC3 (Supplementary Table S2), only PC1 and PC2 were used for examining the geographic patterns of the calls. The 95% confidence ellipses of the groups of individuals from different geographic areas are shown on the plot of PC1 against PC2 (Fig. 3). If the 95% confidence ellipses of the eight geographic areas overlapped, we can treat all the sampled individuals as one population and pool them for further analysis.

The following statistical tests were performed on the untransformed data of each acoustic variable using JMP Pro 14.2.0. First, we treated individuals as our sample units, and we used the averaged measurements from the calls of each individual. We performed Spearman rank tests to examine the relationships between ambient noise levels and each acoustic variable for 65 individuals (because noise measurements were not taken for two individuals). To distinguish variables’ relationship to ambient noise levels, we then classified the 30 acoustic variables into two categories: (1) noise-related variables had a significant relationship with ambient noise levels, and (2) noise-unrelated variables did not. Acoustic variables which had a significant (positive or negative) correlation with ambient noise levels using Spearman rank tests were classified as noise-related variables; all remaining variables lacking such a significant correlation were classified as noise-unrelated variables.

To determine variables which can distinguish the calls of different individuals, we treated calls as sample units and individuals as groups. We then performed a Kruskal–Wallis test to select variables which were more likely to encode individual information, that is, with significant individual differences. We only included those variables with a significant P-value into the DFA (see details below). To investigate how ambient noise levels affected the transmission efficacy of vocal individuality, we used DFA which calculates the accuracy of correctly assigning a particular call to a particular individual. The purpose of a DFA is thus to discriminate sampled individuals based on all the possible acoustic measurements which encode information about individual identity; therefore, we did not perform a variable selection process. Specifically, we used the accuracy value (1—misclassification rate) calculated from the DFA to assess the transmission efficacy of vocal individuality information. Higher accuracy values are assumed to indicate higher transmission efficacy of vocal individuality information from calls recorded through various ambient noise levels.

To compare possible differences in the transmission efficacy of vocal individuality for different sets of acoustic variables, we then calculated a separate DFA for three datasets: (1) all the 30 acoustic variables, (2) the noise-related variables, and (3) the noise-unrelated variables. For each dataset, a separate DFA was used to calculate one overall accuracy value and 67 individual accuracy values. The overall accuracy value (1—misclassification rate) describes the ability of the DFA to correctly assign the 1925 calls to the 67 recorded individuals. The individual accuracy values then describe the ability of the DFA to correctly assign the calls of one particular individual to that individual. To account for small sample sizes of calls by some individuals, the overall misclassification rates were bootstrapped with fractional weights option (number of bootstrap samples, n = 20,000), and the overall accuracy values of the three datasets and the associated 95% biased-corrected confidence intervals (BCI) were obtained. Sixty-seven individual accuracy values (corresponding to the 67 sampled nightjars) were also calculated for each set of variables.

To compare the individual accuracy values using the noise-related variables with the individual accuracy values using the noise-unrelated variables, we used the Wilcoxon signed rank test. To examine how noise levels affected the transmission efficacy through different acoustic structures (noise-related vs. noise-unrelated), we investigated the correlation between the individual accuracy value and the ambient noise level associated with that particular individual by using Spearman rank tests. The differences of the individual accuracy values, which was taken as the accuracy value of the DFA using the noise-unrelated variables minus the accuracy value of the DFA using the noise-related variables from the same individual, were correlated with the ambient noise levels by using Spearman rank tests to examine the similarity in trends.

For the playback-recording experiments, seven sound found files were played in each site (high, medium or low urban noise levels), and seven corresponding recordings were received as seven samples for each site. In each recording sample, although 10 calls of each individual (identified by frequency shift values) were played, the number of received calls might be less than 10 because the calls were overlapped with other unexpected sounds and thus excluded for measurements. Therefore, we first averaged the measurements of the possible received calls for each individual in a sample and then used the averaged measurements as the measurements of the sample. Thus, we obtained 49 samples (7 samples × 7 individuals) for each site for analyzing the overall accuracy of vocal individuality and variable accuracy for the playback-recording experiments. Since we excluded the duration variable, only 29 variables were used for DFA because the duration was fixed in measurements for all individuals. Furthermore, we only used six main frequency-based variables (PFSTART, PFEND, PFMIN, PFMAXA, PFMAX, PFMEAN) for further comparison on transmission accuracy between noise-related variables and noise-unrelated variables because the artificial calls were generated by transforming only the frequency domain. Thus, for each site, we calculated a separate DFA for three datasets: (1) all the 29 acoustic variables, (2) the three noise-related variables (PFSTART, PFEND, PFMIN), and (3) the three noise-unrelated variables (PFMAXA, PFMAX, PFMEAN). For the DFA, the overall misclassification rates were also bootstrapped (number of bootstrap samples, n = 20,000), and the overall accuracy values of the three datasets and the associated 95% biased-corrected confidence intervals (BCI) were obtained. Furthermore, for the six main frequency-based variables, an accuracy value for each variable was calculated as 1 − (|R – B| /B), in which R indicated the measurement value of the received call and B indicated the measurement value of the broadcast call. In each site for each variable, the accuracy values of the seven samples of the same individual were averaged as the variable accuracy value for the individual. In each site, we then investigated the differences of variable accuracy values among variables by Friedman rank tests (with individual as block) and Wilcoxon signed rank test for paired comparison between variables. We set the significance level at 0.05 for all tests and report the two-tailed probability values.

## Supplementary information

Supplementary Legends.

Supplementary Audio.

Supplementary Information 1.
