# Supplementary material for: Acoustic preadaptation to transmit vocal individuality of savanna nightjars in noisy urban environments
Source: Sci Rep. 2020 Oct 23;10:18159. doi: 10.1038/s41598-020-75371-4 (PMC7584573; doi:10.1038/s41598-020-75371-4)
Supplement: Supplementary file 1 — Supplementary Legends. [file 41598_2020_75371_MOESM1_ESM.docx]

Table S1: Descriptive statistics of 30 acoustic variables (n = 67 individuals) plus ambient noise levels (n = 65 sites) (see Table 1 for variable descriptions).

Table S2: Results of the PCA on the 30 normalized variables using the first five principal components (see Table 1 for variable descriptions).

Table S3: Spearman rank tests to test for relationships with ambient noise levels (n = 65 individuals because noise measurements were not taken for two individuals) for 30 acoustic variables using untransformed data (see Table 1 for variable descriptions). Noise-related variables (P < 0.05) are shown into bold italics.

Table S4: Kruskal-Wallis tests (ChiSquare value) to test for individual differences (individual as group, 67 individuals) for 30 acoustic variables using untransformed data (see Table 1 for variable descriptions).

Table S5: Results of Wilcoxon signed rank tests for multiple comparisons among variables of playback-recording experiments in three urban noise levels (see Table 1 for variable descriptions).

Table S6: Descriptions of acoustic measurements for the 7 frequency-shifted calls in playback-recording experiments (see Table 1 for variable descriptions).

Audio S1: A representative call of a savanna nightjar.
