# Supplementary material for: Acoustic preadaptation to transmit vocal individuality of savanna nightjars in noisy urban environments
Source: Sci Rep. 2020 Oct 23;10:18159. doi: 10.1038/s41598-020-75371-4 (PMC7584573; doi:10.1038/s41598-020-75371-4)
Supplement: Supplementary file 3 — Supplementary Information 1. [file 41598_2020_75371_MOESM3_ESM.pdf]

# Acoustic preadaptation to transmit vocal individuality of savanna nightjars in noisy urban environments

Shih-Hsiung Liang[1], Bruno Andreas Walther[2], Chia-Hung Jen[3], Chao-Chieh Chen[4], Yi-Chih Chen[1], Bao-Sen Shieh[4,5*]

[1]Department of Biotechnology, National Kaohsiung Normal University, Kaohsiung 824, Taiwan
[2]Department of Biological Sciences, National Sun Yat-sen University, Kaohsiung 804, Taiwan
[3]Department of Geography, CDTL, National Kaohsiung Normal University, Kaohsiung 802, Taiwan.
[4]Department of Biomedical Science and Environmental Biology, Kaohsiung Medical University, Kaohsiung 807, Taiwan
[5]Department of Medical Research, Kaohsiung Medical University Hospital, Kaohsiung 807, Taiwan

*Corresponding Author:
Bao-Sen Shieh
100 Shihchuan 1st Road, Kaohsiung 807, Taiwan
e-mail address: bsshieh@kmu.edu.tw
Tel: +886-7-3121101ext. 2703
Fax: +886-7-3227508

**Supplementary Materials**

Supplementary Table S1. Descriptive statistics of 30 acoustic variables (n = 67 individuals) plus ambient noise levels (n = 65 sites) (see Table 1 for variable descriptions).

| Variable | Mean | Standard Error of Mean |
|---|---|---|
| Ambient noise level | 80.24 | 0.74 |
| Time-based variables | | |
| DUR | 0.2172 | 0.0019 |
| DISTTOMAX | 0.1357 | 0.0034 |
| Frequency-based variables | | |
| PFSTART | 2871.88 | 74.75 |
| Q1START | 2584.76 | 35.77 |
| Q2START | 3320.57 | 46.17 |
| Q3START | 4231.36 | 45.69 |
| PFEND | 3594.80 | 65.24 |
| Q1END | 2967.26 | 42.53 |
| Q2END | 3712.63 | 35.26 |
| Q3END | 4438.64 | 27.68 |
| PFMAXA | 3892.65 | 58.78 |
| Q1MAXA | 3316.19 | 33.47 |
| Q2MAXA | 3851.46 | 44.10 |
| Q3MAXA | 4253.39 | 46.85 |
| PFMIN | 2275.05 | 28.14 |
| Q1MIN | 2416.20 | 18.15 |
| Q2MIN | 2989.24 | 16.79 |
| Q3MIN | 3523.14 | 17.92 |
| PFMAX | 5348.34 | 53.27 |
| Q1MAX | 3941.83 | 47.49 |
| Q2MAX | 4733.74 | 48.37 |
| Q3MAX | 5290.82 | 50.65 |
| PFMEAN | 3788.59 | 23.59 |
| Q1MEAN | 3161.14 | 24.16 |
| Q2MEAN | 3757.84 | 20.82 |
| Q3MEAN | 4256.90 | 22.38 |
| Frequency-modulation-based variables | | |
| PFSTDDEV | 0.1943 | 0.0040 |
| Q1STDDEV | 0.1099 | 0.0026 |
| Q2STDDEV | 0.1112 | 0.0024 |
| Q3STDDEV | 0.1061 | 0.0021 |

Supplementary Table S2. Results of the PCA on the 30 normalized variables using the first five principal components (see Table 1 for variable descriptions).

| | Principal components | | | | |
|---|---|---|---|---|---|
| | PC1 | PC2 | PC3 | PC4 | PC5 |
| Eigenvalue | 13.7 | 5.99 | 2.81 | 2.67 | 1.12 |
| %Variance | 45.7 | 20 | 9.4 | 8.9 | 3.7 |
| Variables | Coefficient (Eigenvector) | | | | |
| DUR | 0.004 | -0.050 | 0.172 | -0.131 | -0.753 |
| DISTTOMAX | -0.111 | -0.140 | 0.135 | -0.212 | -0.027 |
| PFSTART | -0.166 | 0.223 | -0.10 | 0.220 | -0.181 |
| Q1START | -0.175 | 0.256 | 0.014 | 0.157 | -0.159 |
| Q2START | -0.197 | 0.197 | -0.111 | 0.201 | -0.166 |
| Q3START | -0.204 | 0.050 | -0.218 | 0.202 | -0.027 |
| PFEND | -0.174 | 0.177 | 0.096 | -0.226 | 0.194 |
| Q1END | -0.183 | 0.212 | 0.159 | -0.120 | 0.151 |
| Q2END | -0.216 | 0.157 | 0.015 | -0.148 | 0.198 |
| Q3END | -0.223 | -0.002 | -0.207 | -0.054 | 0.149 |
| PFMAXA | -0.150 | -0.179 | 0.022 | -0.336 | -0.217 |
| Q1MAXA | -0.214 | -0.071 | 0.183 | -0.168 | 0.170 |
| Q2MAXA | -0.181 | -0.181 | 0.049 | -0.276 | -0.150 |
| Q3MAXA | -0.172 | -0.188 | -0.103 | -0.289 | -0.156 |
| PFMIN | -0.149 | 0.307 | 0.089 | 0.072 | -0.137 |
| Q1MIN | -0.168 | 0.291 | -0.011 | 0.081 | -0.122 |
| Q2MIN | -0.193 | 0.197 | -0.205 | -0.040 | -0.087 |
| Q3MIN | -0.122 | -0.037 | -0.445 | -0.217 | 0.078 |
| PFMAX | -0.183 | -0.199 | -0.20 | 0.190 | 0.004 |
| Q1MAX | -0.221 | -0.037 | 0.275 | 0.111 | 0.069 |
| Q2MAX | -0.230 | -0.165 | 0.113 | 0.109 | -0.052 |
| Q3MAX | -0.201 | -0.208 | -0.123 | 0.183 | -0.031 |
| PFMEAN | -0.227 | -0.021 | 0.048 | -0.111 | -0.005 |
| Q1MEAN | -0.234 | 0.109 | 0.195 | -0.027 | 0.100 |
| Q2MEAN | -0.256 | -0.028 | -0.005 | -0.041 | 0.050 |
| Q3MEAN | -0.207 | -0.124 | -0.309 | -0.025 | 0.017 |
| PFSTDDEV | -0.010 | -0.321 | -0.276 | 0.169 | -0.007 |
| Q1STDDEV | -0.154 | -0.228 | 0.274 | 0.146 | 0.178 |
| Q2STDDEV | -0.150 | -0.243 | 0.231 | 0.221 | -0.013 |
| Q3STDDEV | -0.138 | -0.206 | 0.159 | 0.352 | -0.036 |

Supplementary Table S3. Spearman rank tests to test for relationships with ambient noise levels (n = 65 individuals because noise measurements were not taken for two individuals) for 30 acoustic variables using untransformed data (see Table 1 for variable descriptions). Noise-related variables (P < 0.05) are shown into bold italics.

| Variables | Spearman ρ | P-value |
|---|---|---|
| DUR | 0.0086 | 0.9458 |
| DISTTOMAX | 0.029 | 0.8187 |
| ***PFSTART*** | ***-0.468*** | ***<.0001*** |
| ***Q1START*** | ***-0.6108*** | ***<.0001*** |
| ***Q2START*** | ***-0.5223*** | ***<.0001*** |
| ***Q3START*** | ***-0.3992*** | ***0.001*** |
| ***PFEND*** | ***-0.3636*** | ***0.0029*** |
| ***Q1END*** | ***-0.615*** | ***<.0001*** |
| ***Q2END*** | ***-0.5334*** | ***<.0001*** |
| ***Q3END*** | ***-0.3049*** | ***0.0135*** |
| PFMAXA | 0.1231 | 0.3285 |
| Q1MAXA | -0.2091 | 0.0946 |
| Q2MAXA | 0.0117 | 0.9262 |
| Q3MAXA | 0.0562 | 0.6565 |
| ***PFMIN*** | ***-0.5341*** | ***<.0001*** |
| ***Q1MIN*** | ***-0.5876*** | ***<.0001*** |
| ***Q2MIN*** | ***-0.3933*** | ***0.0012*** |
| Q3MIN | 0.0619 | 0.6245 |
| PFMAX | -0.0275 | 0.8279 |
| ***Q1MAX*** | ***-0.4219*** | ***0.0005*** |
| Q2MAX | -0.2019 | 0.1068 |
| Q3MAX | -0.0329 | 0.7946 |
| PFMEAN | -0.1677 | 0.1818 |
| ***Q1MEAN*** | ***-0.5159*** | ***<.0001*** |
| ***Q2MEAN*** | ***-0.3023*** | ***0.0144*** |
| Q3MEAN | -0.0897 | 0.4774 |
| ***PFSTDDEV*** | ***0.3214*** | ***0.009*** |
| Q1STDDEV | -0.0847 | 0.5023 |
| Q2STDDEV | -0.0904 | 0.4741 |
| Q3STDDEV | -0.1062 | 0.3999 |

Supplementary Table S4. Kruskal-Wallis tests (ChiSquare value) to test for individual differences (individual as group, 67 individuals) for 30 acoustic variables using untransformed data (see Table 1 for variable descriptions).

| Variables | ChiSquare | P-value |
|---|---|---|
| DUR | 1637.1 | <.0001 |
| DISTTOMAX | 468.1 | <.0001 |
| PFSTART | 393.2 | <.0001 |
| Q1START | 677.4 | <.0001 |
| Q2START | 661.1 | <.0001 |
| Q3START | 854.4 | <.0001 |
| PFEND | 493.5 | <.0001 |
| Q1END | 1243.9 | <.0001 |
| Q2END | 1083.1 | <.0001 |
| Q3END | 1039.7 | <.0001 |
| PFMAXA | 582.8 | <.0001 |
| Q1MAXA | 720.8 | <.0001 |
| Q2MAXA | 648.9 | <.0001 |
| Q3MAXA | 642.5 | <.0001 |
| PFMIN | 607.6 | <.0001 |
| Q1MIN | 783.5 | <.0001 |
| Q2MIN | 832.8 | <.0001 |
| Q3MIN | 1327.8 | <.0001 |
| PFMAX | 1414.7 | <.0001 |
| Q1MAX | 1068.0 | <.0001 |
| Q2MAX | 1183.4 | <.0001 |
| Q3MAX | 1538.6 | <.0001 |
| PFMEAN | 1138.8 | <.0001 |
| Q1MEAN | 1335.1 | <.0001 |
| Q2MEAN | 1316.4 | <.0001 |
| Q3MEAN | 1573.8 | <.0001 |
| PFSTDDEV | 1096.9 | <.0001 |
| Q1STDDEV | 995.4 | <.0001 |
| Q2STDDEV | 1076.8 | <.0001 |
| Q3STDDEV | 1413.6 | <.0001 |

Supplementary Table S5. Results of Wilcoxon signed rank tests for multiple comparisons among variables of playback-recording experiments in three urban noise levels (see Table 1 for variable descriptions).

| | Urban Noise Levels | | | | | |
|---|---|---|---|---|---|---|
| | Low | | Medium | | High | |
| Comparison | Test Statistics | *P* | Test Statistics | *P* | Test Statistics | *P* |
| PFMAXA-PFEND | 7 | 0.2969 | 6 | 0.375 | 11 | 0.0781 |
| PFMAX-PFEND | 10 | 0.1094 | 14 | 0.0156 | 9 | 0.1563 |
| PFMAX-PFMAXA | 4 | 0.5781 | 5 | 0.4688 | -8 | 0.2188 |
| PFMEAN-PFEND | 10 | 0.1094 | 3 | 0.6875 | 14 | 0.0156 |
| PFMEAN-PFMAXA | 1 | 0.9375 | -6 | 0.375 | 0 | 1 |
| PFMEAN-PFMAX | -7 | 0.2969 | -14 | 0.0156 | 13 | 0.0313 |
| PFMIN-PFEND | -10 | 0.1094 | -8 | 0.2188 | 14 | 0.0156 |
| PFMIN-PFMAXA | -9 | 0.1563 | -7 | 0.2969 | -2 | 0.8125 |
| PFMIN-PFMAX | -14 | 0.0156 | -14 | 0.0156 | 12 | 0.0469 |
| PFMIN-PFMEAN | -12 | 0.0469 | -4 | 0.5781 | -3 | 0.6875 |
| PFSTART-PFEND | -14 | 0.0156 | -14 | 0.0156 | 9 | 0.1563 |
| PFSTART-PFMAXA | -14 | 0.0156 | -14 | 0.0156 | -12 | 0.0469 |
| PFSTART-PFMAX | -14 | 0.0156 | -14 | 0.0156 | 0 | 1 |
| PFSTART-PFMEAN | -14 | 0.0156 | -14 | 0.0156 | -8 | 0.2188 |
| PFSTART-PFMIN | -14 | 0.0156 | -14 | 0.0156 | -9 | 0.1563 |

Supplementary Table S6. Descriptions of acoustic measurements for the 7 frequency-shifted calls in playback-recording experiments (see Table 1 for variable descriptions).

| | Frequency-shift treatment | | | | | | |
|---|---|---|---|---|---|---|---|
| Variable | -300 | -200 | -100 | 0 | +100 | +200 | _+300 |
| DUR | 0.2266 | 0.2266 | 0.2266 | 0.2266 | 0.2266 | 0.2266 | 0.2266 |
| DISTTOMAX | 0.2051 | 0.2044 | 0.2046 | 0.2051 | 0.2044 | 0.2046 | 0.2051 |
| PFSTART | 2020 | 2138 | 2226 | 2320 | 2442 | 2535 | 2620 |
| Q1START | 2135 | 2220 | 2330 | 2435 | 2527 | 2625 | 2727 |
| Q2START | 2635 | 2774 | 2862 | 2950 | 3036 | 3150 | 3246 |
| Q3START | 3315 | 3422 | 3566 | 3620 | 3771 | 3867 | 3924 |
| PFEND | 4424 | 4480 | 4572 | 4724 | 4784 | 4872 | 5021 |
| Q1END | 3755 | 3852 | 3961 | 4062 | 4156 | 4261 | 4361 |
| Q2END | 4385 | 4490 | 4588 | 4686 | 4792 | 4884 | 4986 |
| Q3END | 4630 | 4740 | 4828 | 4934 | 5032 | 5128 | 5226 |
| PFMAXA | 3350 | 3480 | 3570 | 3660 | 3780 | 3870 | 3960 |
| Q1MAXA | 3440 | 3530 | 3635 | 3740 | 3830 | 3930 | 4040 |
| Q2MAXA | 3640 | 3740 | 3834 | 3930 | 4040 | 4134 | 4236 |
| Q3MAXA | 4083 | 4170 | 4304 | 4383 | 4470 | 4601 | 4690 |
| PFMIN | 2020 | 2138 | 2226 | 2320 | 2442 | 2535 | 2620 |
| Q1MIN | 2135 | 2220 | 2330 | 2435 | 2522 | 2621 | 2727 |
| Q2MIN | 2549 | 2688 | 2775 | 2872 | 2963 | 3071 | 3159 |
| Q3MIN | 3105 | 3229 | 3359 | 3422 | 3571 | 3659 | 3718 |
| PFMAX | 6394 | 6500 | 6580 | 6690 | 6800 | 6890 | 6982 |
| Q1MAX | 4073 | 4166 | 4272 | 4365 | 4466 | 4568 | 4678 |
| Q2MAX | 4608 | 4710 | 4804 | 4903 | 5006 | 5108 | 5202 |
| Q3MAX | 5940 | 6026 | 6118 | 6254 | 6338 | 6406 | 6559 |
| PFMEAN | 3815 | 3883 | 3980 | 4106 | 4188 | 4279 | 4399 |
| Q1MEAN | 3272 | 3371 | 3470 | 3567 | 3670 | 3770 | 3868 |
| Q2MEAN | 3774 | 3875 | 3974 | 4073 | 4175 | 4273 | 4372 |
| Q3MEAN | 4221 | 4319 | 4419 | 4522 | 4620 | 4719 | 4822 |
| PFSTDDEV | 0.1645 | 0.1667 | 0.1633 | 0.1497 | 0.1549 | 0.1539 | 0.1397 |
| Q1STDDEV | 0.1177 | 0.1141 | 0.1114 | 0.1076 | 0.1048 | 0.1025 | 0.0998 |
| Q2STDDEV | 0.1079 | 0.1043 | 0.1019 | 0.0991 | 0.0974 | 0.0949 | 0.0924 |
| Q3STDDEV | 0.1212 | 0.1181 | 0.1149 | 0.1132 | 0.1103 | 0.1074 | 0.1062 |
